# Intellectual capability and its association with severe dental caries treatment needs in young Israeli adults: a cross-sectional record-based study

**DOI:** 10.1186/s13584-025-00680-w

**Published:** 2025-05-01

**Authors:** Dan Henry Levy, Nirit Yavnai, Joe Ben Itzhak, Yafit Hamzani, Shlomo Paul Zusman, Michael Solomonov

**Affiliations:** 1 Department of Endodontics, Israel Defense Forces (IDF) Medical Corps, Tel Hashomer, Israel; 2 Bina Program, Faculty of Dental Medicine, Hebrew University, Jerusalem, Israel; 3 Department of Community Dentistry, Faculty of Dental Medicine, Hebrew University, Jerusalem, Israel; 4 Oral and Maxillofacial Surgery, Private Clinic, Tel Aviv, Israel; 5 School of Medicine and Dentistry, University of Rochester, New York, USA

**Keywords:** Intellectual capability, Socioeconomic strata, Treatment needs, Root Canal treatment, Tooth extractions

## Abstract

**Background:**

Disparities in dental health resulting from social and economic inequality are a pressing public health concern. Poor and vulnerable populations bear a higher burden of caries exacerbated by limited access to quality dental care. Recent publications have suggested a possible association between intellectual capability and caries risk, as well as lower compliance with publicly funded healthcare services among populations with lower educational levels. The objective of this study was to explore potential associations between severe dental caries treatment needs (root canal treatments and extractions), socioeconomic factors and intellectual capability.

**Methods:**

Dental records of 21,052 soldiers recruited into Israeli military service between 2019 and 2021 were reviewed, and data on the need for root canal treatments and extractions were retrieved. Sociodemographic information, including age, sex, socioeconomic strata, intellectual capability scores, body mass index, and place of birth, were extracted and analyzed.

**Results:**

The findings from the multivariate generalized linear model indicated that lower intellectual capability scores and socioeconomic strata were associated with a significantly greater requirement for root canal treatments and extractions (*p* < 0.001). The model also identified male gender and older age as predictors for higher treatment needs. Non-native Israelis were found to be at a greater risk for needing root canal treatments compared to native Israelis. Additionally, a significant positive correlation was observed between intellectual capability scores and socioeconomic strata (*p* < 0.001).

**Conclusions:**

Groups with lower intellectual capability scores and socioeconomic strata exhibited a greater need for dental treatments, including root canal treatments and extractions. Given their higher likelihood of requiring more invasive treatments, health policy should prioritize intervention plans aimed at improving attendance at preventive care services for these disadvantaged populations under the Israeli free dental care reform.

## Background

The prevalence of dental caries remains high worldwide, affecting billions of people. It is on the rise due to the growing global population, extended longevity, and the increase of risk factors, such as free sugar consumption and other habits deleterious to oral health [1]. Caries is known to be more prevalent in poor and vulnerable groups in society [2], often resulting in deep lesions that lead to the need for more complex management, such as root canal therapy (RCT) or extractions. Unfortunately, the decision of whether to perform RCT to preserve the tooth or whether to extract it, is not solely based upon clinical considerations. Socioeconomic and racial factors reportedly also play a role, with several studies having shown that populations with lower socioeconomic status are more likely to undergo tooth extractions instead of RCT [3, 4]. A higher prevalence of dental caries has also been observed in populations with lower intellectual capability and less education [5, 6].

The objective of this study was to utilize a vast database of otherwise fit young Israeli male and female adults who underwent a comprehensive screening process prior to being inducted into the army, as well as dental examinations shortly after their recruitment in order to investigate the association between intellectual capability score (ICS) and socioeconomic strata (SES) with severe consequences of neglected caries, specifically, the need for RCTs and extractions. As there is universal health coverage for dental treatment prior to the enrolment, such information might be relevant to fine tune the public policy and assure differential care according to the needs of this population.

## Methods

The data for this study included findings on dental records, sociodemographic characteristics, and intellectual screening results of 21,052 young adults recruited into military service in the IDF between 2019 and 2021. Data on dental treatment needs were digitally collected from dental treatment plans that were part of the mandatory medical examinations in basic training bases during the first 4 months post-induction. This data included the number of required restorations, number of teeth in need of RCT, and number of extractions needed due to caries.

All dental treatment plans were made after a clinical examination and two bitewing radiographs. Appropriate periapical radiographs were also taken in cases of deep caries or earlier endodontic treatment. All subjects were otherwise healthy, having been found medically fit for combat military service. All dentists who performed the examinations had similar levels of dental experience and had undergone periodic quality assurance evaluations by regional dental officers.

Dental data were crossed-matched with the following independent sociodemographic variables: age, sex, whether born in Israel or elsewhere, SES, and ICS. SES of each subject was determined by socioeconomic level of his/her place of residence ranked by the Israel Bureau of Statistics (1 being the lowest and 10 the highest SES) [7]. Every potential recruit underwent a routine psycho-technical evaluation ranked on a 9-point scale in order to establish their ICS prior to induction. That evaluation is comprised of four tests: [1] a modified Otis-type verbal intelligence test designed to assess the subject’s ability to understand and execute verbal instructions; [2] verbal categorization and abstraction tests that measure verbal analogies and are based upon a “similarities” subtest of the Wechsler Intelligence Scales; [3] a mathematics ability test, measuring mathematical reasoning, concentration, and concept manipulation; [4] a nonverbal analogies test based upon Raven’s Progressive Matrices that evaluate nonverbal abstract reasoning and visual–spatial problem-solving abilities. These tests have a correlation of > 0.8 with the classic Wechsler Adult Intelligence Scale [8, 9]. ICS have been used as a proxy for the intelligence quotient (IQ) in the IDF, as reported elsewhere [10, 11].

Statistical analyses were performed using SPSS software (version 28). The significance level was set at *p* < 0.05. Univariate analyses included descriptive statistics by means and standard deviations (SDs) for continuous variables and distributions by percentages for categorical variables. Means comparisons were performed with Mann-Whitney and Kruskal-Wallis tests. Spearman correlation coefficients were calculated for linear correlations. A multivariate general linear model (GLM) was built and included statistically significant independent variables (e.g., sociodemographic variables) and other variables for adjustment in order to predict severe dental caries and dental treatment needs.

## Results

The analysis included a total of 21,052 records of recruits with a mean age of 18.98 years (range 17.69–28.48, SD = 0.83), of whom 90.65% were males. The mean body mass index (BMI) of the cohort was 22.32 (SD = 3.36). and the median ICS was 50 (range 10–90). Most (92.23%) of the subjects were born in Israel. The mean number of teeth in need of restorations was 1.56 per subject (SD = 2.24), 0.06 (SD = 0.31) for RCTs and 0.04 (SD = 0.25) for extractions.

Associations between higher treatment needs and lower ICS and SES, as well as between ICS, SES, BMI, age, and treatment needs are presented in Table 1. Associations between male sex and place of birth being other than Israel and higher treatment needs are shown in Table 2. The GLM revealed that male sex, older age, low ICS, and low SES were all predictors for higher treatment needs, including restorations, RCTs, and extractions (Tables 3 and 4). A lower BMI was significantly associated with more teeth in need of extraction (*p* = 0.04) but not with other treatment needs. Not being born in Israel was found to be a risk factor, with a higher number of teeth in need of RCT compared to Israeli-born subjects (odds ratio = 1.023, *p* = 0.005, Table 3). Finally, there was a significant and direct linear correlation between ICS and SES (Pearson correlation coefficient = 0.19, *p* < 0.001).

**Table 1:** Correlation between ICS, SES, BMI, age and number of teeth with caries-related treatment needs

Parameter	*N*	Number of Teeth in Need of Restorations		Number of Teeth in Need of RCT		Number of Teeth in Need of Extractions	
		Correlation Coefficient	*p*-Value*	Correlation Coefficient	*p*-Value*	Correlation Coefficient	*p*-Value*
ICS	20,545	-0.161	< 0.001	-0.111	< 0.001	-0.115	< 0.001
SES	18,829	-0.153	< 0.001	-0.088	< 0.001	-0.089	< 0.001
BMI	21,040	0.002	0.751	-0.010	0.162	-0.017	0.014
Age	21,052	0.049	< 0.001	0.022	0.001	0.027	< 0.001

*ICS*: Intellectual capability score; *SES*: Socioeconomic strata; *BMI*: Body mass index; *RCT*: Root canal treatment

*Spearman Correlation Test

**Table 2:** Association between sex, birthplace and number of teeth with caries-related treatment needs

		*N*	Number of Teeth in Need of Restorations		Number of Teeth in Need of RCT		Number of Teeth in Need of Extractions	
			Mean (SD)	*p-*Value^a^	Mean (SD)	*p*-Value^a^	Mean (SD)	*p-*value^*^
Sex	Female	1,968	1.131 (1.9129)	< 0.001	0.340 (0.2712)	< 0.001	0.02 (0.152)	0.001
	Male	19,084	1.592 (2.2634)		0.566 (0.3103)		0.04 (0.257)	
Birthplace	Israel	19,417	1.544 (2.2336)	0.282	0.0530 (0.3036)	0.009	0.04 (0.249)	0.572
	Other	1,635	1.603 (2.27613)		0.0722 (0.344)		0.04 (0.246)	
	Total	21,052	1.556 (2.237)		0.055 (0.307)		0.04 (0.249)	

*RCT:* Root canal treatment.

^*^Mann-Whitney Test

**Table 3:** Generalized linear model for the number of teeth requiring RCT as the independent variable (*p* < 0.001)

Variable		B	*p*-Value	Exp(B)	Lower	Upper
Intercept		0.087	0.116	1.091	0.979	1.216
Sex	Female	-	-	1	-	-
	Male	0.016	0.04	1.016	1.001	1.031
Birthplace	Israel	-	-	1	-	-
	Other	0.023	0.005	1.023	1.007	1.039
SES		-0.012	< 0.001	0.988	0.985	0.991
ICS		-0.002	< 0.001	0.998	0.998	0.999
BMI		-0.001	0.256	0.999	0.998	1.001
Age		0.007	0.017	1.007	1.001	1.012

*ICS*: Intellectual capability score; *SES*: Socioeconomic strata; *BMI*: Body mass index; *RCT*: Root canal treatment

**Table 4:** Generalized linear model for the number of teeth requiring extractions as the independent variable (*p* < 0.001)

Variable		B	*p*-Value	Exp(B)	Lower	Upper
Intercept		0.046	0.314	1.047	0.957	1.145
Sex	Female	-	-	1	-	-
	Male	0.012	0.05	1.013	1.000	1.025
Birthplace	Israel	-	-	1	-	-
	Other	-0.001	0.930	0.999	0.986	1.013
SES		-0.009	< 0.001	0.991	0.989	0.993
ICS		-0.001	< 0.001	0.999	0.998	0.999
BMI		-0.002	0.04	0.998	0.997	1.000
Age		0.008	0.001	1.008	1.003	1.012

*ICS*: Intellectual capability score; *SES*: Socioeconomic strata; *BMI*: Body mass index

## Discussion

Dental caries is the most prevalent health condition worldwide [2]. In recent years, Israel has worked to improve oral health among underprivileged populations by implementing legislation that provides free dental care for children and adolescents from birth to 18 years of age through a child dental care reform (CDCR) under the National Health Insurance Law (NHIL). A study on the utilization levels of the CDCR by Domb Herman et al. concluded that despite a significant increase in the percentage of children using the services from 8 to 33% with the inclusion of additional age groups, more efforts are needed to boost utilization [12]. However, a recent study by Levy et al. showed that the provision of free dental care is not enough to reduce the overall dental treatment needs among Israeli youth [13], while it is enough to lower the untreated carious lesions in Israeli 6-year-olds, as Natapov et al. found [14]. These findings highlight the need for decision-makers to implement strategies within dental health policies to further improve attendance rates.

The current analysis demonstrated a significant (*p* < 0.001) inverse correlation between ICS and the need for dental restorations, RCTs and extractions, even after adjusting for confounding factors, such as age, sex, BMI, and country of birth, in line with the findings reported in the literature [5, 15, 16].

ICS can influence dental treatment needs in multiple ways. For example, lower ICS may lead to less awareness of dental health, resulting in poorer dental hygiene habits, such as inadequate tooth brushing and infrequent use of dental floss [15, 17], leading to more dental plaque and periodontal disease [15, 18]. Lower levels of parental education and patient ICS were also reportedly associated with greater consumption of cariogenic processed foods and sweetened beverages [4, 5, 19]. Recently, Khatib et al. [20] found that a low educational level hinders Israeli Arab children from utilizing dental services covered under Israel’s NHIL. According to the authors, this barrier arises because individuals with lower educational attainment require more explicit information about new services [20]. Taken together, it can be suggested that low ICS and a low educational level not only contribute to the risk of caries but also impede effective treatment uptake.

Moreover, there was a direct association between lower ICS and SES in the current study cohort, raising the possibility that ICS may indirectly contribute to an increase in caries experience attributed to low SES [21]. Low income and educational levels both contribute to poor health literacy, which negatively impacts self-assessed health, a common indicator of overall health [22]. Children from lower SES backgrounds are reportedly more likely to attend dental clinics for urgent treatments and less likely to attend them for routine check-ups compared to children from higher SES groups [4, 23]. This highlights the need to enhance dental care in underserved areas, addressing both resource limitations and factors such as educational levels and ICS. Implementing intervention plans, such as supervised tooth brushing programs (STBPs) in kindergartens, could help reduce dental health disparities, as evidenced by their effectiveness in low SES areas of Israel [24].

The recruits in the current study who were immigrants to Israel needed significantly more RCTs compared to native Israelis (*p =* 0.009). Immigration often involves a change to a more cariogenic diet, resulting in an increase in caries experience [25]. Immigrant children in Israel may have had limited access to free dental care, depending on their age of arrival, as well as to school dental services (SDS), which could result in suboptimal dental care. Evidence suggests that most immigrants only sought dental care when experiencing pain, in contrast to Israeli children, who were more likely to receive regular dental check-ups [26]. In some communities, higher rates of dental caries have been observed not only among the first generation of immigrants but also in subsequent generations [11], Some studies suggest an association with the father’s lower professional occupation [26], while others emphasize the role of psychological distress and social support among immigrants [27].

Globally, oral health care is less accessible to less educated populations [6, 28], people from disadvantaged municipalities [29], people of certain ethnic backgrounds [3], children with no dental health insurance [30], and individuals in lower SES groups [2]. Unfortunately, they comprise the groups with more prevalent dental caries [31]. In fact, the cost of dental treatment in certain countries can be so significant that it can push families below the poverty line [32]. Several studies have shown that dentists tend to offer less privileged patients more invasive treatments, such as tooth extractions, over more conservative approaches that tend to be more costly [3, 4]. Patel et al. recently showed that dentists were more likely to offer RCT to a White patient and extraction to a Black patient [33]. Similar studies in different armies have revealed comparable findings. For example, studies in the Finnish army found an association between lower caries-related treatment needs and factors such as better education and socioeconomic status, water fluoridation, physical fitness, living in urban areas, and reduced sugar consumption. However, the studies concluded that the health behavior of young Finnish males does not support good oral health, making caries control an ongoing challenge [34–37]. In a similar vein, in the United States Air Force, while education and sociodemographic factors still influenced caries risk, the importance of prevention programs and caries risk assessment was strongly emphasized in reducing smoking and caries prevalence [38].

There was also a significantly greater need for dental restorations among the current recruits who belonged to populations with lower SES and lower ICS (*p* < 0.001, Table 1), suggesting that further tooth loss can be anticipated without the implementation of significant intervention. In fact, the timing of dental checkups and preventive care appointments is crucial in treating caries, given its progressive nature. Even if a root canal treatment is needed, performing it on time can prevent the progression to tooth extraction. It has been indicated that, although dental care in the army is free and accessible, demand remains influenced by social background. Untreated caries at the time of discharge is still higher among IDF soldiers with less than 12 years of education [39].

Water fluoridation has been demonstrated as the most effective and widespread method for promoting community-level oral health and addressing dental health inequalities stemming from socioeconomic disparities [40]. Regrettably, legislation enacted in 2014 banned water fluoridation in Israel, halting its beneficial effects and already demonstrating an increase in dental caries levels among Israeli children and in dental disparities [41]. Therefore, the continuation of nationwide water fluoridation is vital for addressing the aforementioned dental health challenges arising from lower SES and ICS.

In the IDF, soldiers from low socioeconomic backgrounds and immigrants without parents in Israel receive a broader range of treatments, including dental crowns and implants, compared to soldiers without financial issues. Based on our findings, it may be advisable to update this policy, for example, by including populations with low ICS in future dental intervention plans, while ensuring the approach remains discreet and sensitive. A preventive approach could be to implement a mandatory periodic dental examination for Israeli military personnel, similar to the required periodic medical examinations, as suggested by Zadik et al. [42]. On the national level, since individuals with lower educational levels and immigrants tend to use dental care services less frequently [12, 20], it is crucial to make information more accessible and clearer. For example, sending letters to parents from the school dental health service advising them that their child needs to see a dentist could help address this issue [23]. Engaging healthcare providers beyond dentists, such as pediatricians and public health nurses, can also help effectively reach these groups [12]. Additionally, initiatives like the recently launched “Smiles” oral health promotion program for preschool children, which involves the support of teaching staff and parents, have shown promising results [43].

Strength and limitations: the current study population is well suited for assessing the association between dental health, SES, and ICS because it is comprised solely of otherwise healthy young adults, thereby eliminating the potential confounding effects of chronic or systemic illnesses. Importantly, dental examinations are mandatory for IDF recruits, enabling the acquisition of data from the entire study population.

There are several limitations to this study. Firstly, the study had a low proportion of women (9%, *N* = 1,968), making it difficult to generalize any findings on sex differences to the wider population. Secondly, cariogenic behaviors, such as high sugar intake and oral hygiene practices, were not considered in this study. Given that specific cariogenic foods and confections can increase the treatment needs of military personnel [44], it is recommended that future research incorporate these factors. Thirdly, the methodology did not include the collection of data on former caries experience, such as filled or missing teeth, but rather focused solely upon treatment needs at the time of the examination.

## Conclusions

Young Israeli recruits with lower intellectual capacity and poor socioeconomic backgrounds have a greater need for invasive dental treatments, such as root canal treatments and extractions. Health policies should prioritize tooth-conserving options by promoting preventive strategies, such as water fluoridation, and by improving access to preventive care.

Additionally, enhancing the accessibility and clarity of dental care information is essential to ensure that individuals from disadvantaged groups can better utilize available resources and prevent severe dental pathologies.

## Data Availability

The datasets generated and/or analyzed during the current study are not publicly available due to IDF data regulations.

## Abbreviations

RCTs: Root canal treatments
SES: Socio-economic strata
ICS: Intellectual capability scores
IDF: Israeli Defense Forces
SDS: School dental service
NHIL: National Health Insurance Law
CDCR: Child Dental Care Reform
STBP: Supervised tooth brushing program
SDs: Standard deviations
GLM: Multivariate general linear model
BMI: Mean body mass index
